# Methadone or buprenorphine: which is the better analgesic for feline ovariohysterectomy?

**DOI:** 10.18849/ve.v9i4.704

**Published:** 2024-11-12

**Authors:** Isobel Lawrence

**Affiliations:** University of Liverpool, United Kingdom

**Keywords:** analgesia, buprenorphine, cat, feline, methadone, ovariohysterectomy, pain, spay

## Abstract

**PICO question:**

In female cats undergoing routine ovariohysterectomy, is using methadone, compared to buprenorphine, in the anaesthetic protocol associated with lower postoperative pain scores?

**Clinical bottom line:**

**Category of research:**

Treatment.

**Number and type of study designs reviewed:**

Four assessor-blinded randomised controlled trials.

**Strength of evidence:**

Moderate.

**Outcomes reported:**

Two studies found methadone to be associated with significantly lower postoperative pain scores than buprenorphine; one showed significantly different pain scores over the entire 8-hour postoperative period and one only at 10 minutes postextubation. One study found buprenorphine to be associated with lower postoperative pain scores at some time points postsurgery. One study found no significant difference in pain scores between methadone and buprenorphine.

**Conclusion:**

It can be concluded that using methadone in the anaesthetic protocol may provide better analgesia for cats undergoing ovariohysterectomy compared to buprenorphine. The two papers that used validated pain scoring both concluded that methadone was associated with lower pain scores at some points postoperatively. Although one of the studies concluded that buprenorphine is associated with lower postoperative pain scores, this paper provides only very weak evidence because an unvalidated pain scoring method was used. More evidence is needed to confidently recommend using one opioid over another.

## How to apply this evidence in practice

The application of evidence into practice should take into account multiple factors, not limited to: individual clinical expertise, patient’s circumstances and owners’ values, country, location or clinic where you work, the individual case in front of you, the availability of therapies and resources.

Knowledge Summaries are a resource to help reinforce or inform decision-making. They do not override the responsibility or judgement of the practitioner to do what is best for the animal in their care.

## Clinical scenario

Feline ovariohysterectomies are some of the most commonly performed surgeries in first opinion veterinary practice. These may be owned animals, but they may also be CNVR (catch, neuter, vaccinate, release) situations where the cat will not be given any further analgesia after discharge; therefore, it is imperative that perioperative analgesia is effective. Opioids are often used in the anaesthetic protocol because they provide reliable analgesia, can be reversed, and have a wide safety margin in cats. However, the choice of opioid often varies between clinicians.

Historically, pain in cats has been undertreated due to signs of pain being difficult to identify in this species, and the ability of cats to mask signs of pain (Steagall & Monteiro, 2019; Steagall et al., 2022). This is an issue because inadequate management of acute, perioperative pain can lead to chronic pain and central sensitisation (Monteiro et al., 2023). The development of several validated feline pain scales, including the UNESP-Botucatu multidimensional composite pain scale (Brondani et al., 2013), the Glasgow Feline Composite Measure Pain Scale (CMPS-F) (Reid et al., 2017), and the Feline Grimace Scale (Evangelista et al., 2019), have allowed us to better assess pain in cats, and are commonly used in the studies that this Knowledge Summary evaluates.

Methadone, buprenorphine, and butorphanol are the most commonly used opioids in feline anaesthetic protocols. Butorphanol has a short duration of action and provides mild analgesia, insufficient for surgical procedures (Warne et al., 2014). This Knowledge Summary investigates the difference in postoperative pain scores when methadone is used in the anaesthetic protocol for routine ovariohysterectomy, compared to when buprenorphine is used. Methadone is a synthetic mu agonist which is a racemic mixture of d and l enantiomers. The d enantiomer also has effects on NMDA (*N*-methyl-d-aspartate) receptors providing an alternative analgesic pathway. Buprenorphine is a semi-synthetic partial μ-opoid agonist (Bortolami & Love, 2015). In the clinical setting this Knowledge Summary will help the clinician decide whether to use methadone or buprenorphine to provide the most reliable analgesia for our feline patients.

## The evidence

A search of the literature returned four studies that were relevant to this PICO. All four studies are assessor-blinded randomised controlled trials directly comparing postoperative pain scores between methadone and buprenorphine.

Although all studies evaluated are randomised controlled trials and therefore are at the same level on the hierarchy of evidence, there is variation in the strength of evidence provided by each study. The study by Shah et al. (2019) provides the strongest evidence as the sample size exceeds the power calculation, validated pain scoring methods are used, and cats were evaluated for a suitable amount of time postoperatively. The study by Mahdmina et al. (2020) provides a weaker level of evidence because the sample size does not meet the target set by the power calculation and cats were only evaluated for 30 minutes postextubation; however, it also used validated pain scoring methods. The studies by Bortolami et al. (2013) and Slingsby et al. (2014) provide the weakest evidence because they use unvalidated pain scoring methods and the sample size when just looking at female cats is too small. Overall, the strength of evidence is moderate.

## Summary of the evidence

**Bortolami et al. (2013):** 

Population	Healthy cats admitted for routine castration and ovariohysterectomy, aged > 5 months, ASA (American Society of Anesthesiologists) classification 1 or 2.
Sample Size	45 cats, 24 females, 21 males. Information not relevant to the PICO will not be followed up on.
Intervention Details	Cats were admitted the night before the procedure, housed in individual kennels, and fasted. Cats were randomly assigned to receive acepromazine and one of either methadone, buprenorphine, or butorphanol. 15/45 cats received acepromazine 0.05 mg/kg and 0.02 mg/kg buprenorphine intramuscular (IM). 15/45 cats received acepromazine 0.05 mg/kg and 0.5 mg/kg methadone. 15/45 cats received acepromazine 0.05 mg/kg and 0.4 mg/kg butorphanol. Cats were left for 30 minutes before intravenous (IV) catheter placement in the cephalic vein. Alfaxalone was given to effect for induction. Cats underwent laryngeal application of lidocaine, and an endotracheal tube was placed. Anaesthesia was maintained with isoflurane in oxygen provided via a T-piece system. Cats underwent castration or flank ovariohysterectomy. If pain scoring deemed it necessary, rescue analgesia was given. All cats received further analgesia of 0.5 mg/kg IM methadone 6 hours after premedication, and 0.2 mg/kg meloxicam was given subcutaneously 8 hours after premedication.
Study design	Blinded, randomised, controlled trial.
Outcome studied	Assessment of pain was carried out subjectively through interactive visual analogue scale (IVAS) pain scoring. This was done before premedication, just before IV catheterisation and at 1.5, 2, 3, 4, 5, 6, 7, 8 and 20–24 hours postoperatively. This was measured by a single, blinded assessor. This involved observation of the cat from outside the kennel, reaction to opening of the kennel door, reaction to speaking, and finally handling. A score of 0–100 mm was given where 0 mm was no pain and 100 mm was the worst possible pain. The result of the IVAS pain scoring determined the need for rescue analgesia. A score ≥ 50 mm indicated the need for rescue analgesia. Intraoperative measurements of heart rate (HR), respiratory rate (RR), end tidal CO_2_ (ETCO_2_) and inspired/expired CO_2_ were taken continuously by a multiparameter and recorded every 5 minutes. Indirect measurements of blood pressure and oxygen saturation were also performed. Level of sedation was assessed using a simple descriptive scale (SDS) and visual analogue scale (VAS) and measured before premedication, every 5 minutes following premedication for 30 minutes, just before IV catheterisation, and at 1.5, 2, 3, 4, 5, 6, 7, 8 and 20–24 hours after premedication. Body temperature, HR, RR, and mechanical nociceptive threshold (MNT) testing were also recorded at each of these time points. These assessments were performed by a single, blinded assessor.
Main findings (relevant to PICO question)	Pain scores were generally acceptably low at all time points for cats in both the methadone and buprenorphine groups. There was no significant difference between IVAS scores uncorrected for rescue analgesia between the buprenorphine and methadone groups at any time point. 1/8 female cats in the buprenorphine group compared to 2/8 female cats in the methadone group required rescue analgesia. However, there was no significant difference between groups for requirement of rescue analgesia.
Limitations	Mixed population of cats of different ages and weights. It is not stated in the paper whether the surgeon was the same for all cats. A difference in surgical technique could have altered postoperative pain. The lack of statistically significant difference in IVAS score between groups could be because IVAS is not validated, and even when using the same assessor there will be variability and observer bias. It must be noted that at the time of publication of this paper, there were no feline validated pain scoring systems available. It was determined that the sample size needed for this study would be 10 cats per group which when combining male and female cats was met. However, there were only 8 females in each group which makes the paper underpowered for this Knowledge Summary.

Table note...

**Mahdmina et al. (2020):** 

Population	Healthy entire female cats recruited from the RSPCA Greater Manchester animal hospital assigned American society of anesthesiologists (ASA) grade 1 or 2 and Body condition score (BCS) < 6/9.
Sample Size	51 cats.
Intervention Details	Nine cats underwent euthanasia during anaesthesia as they were unsuitable for rehoming, and one cat did not receive atipamezole at the correct time so was excluded. 41 cats underwent surgery and pain scoring. Cats were starved for 12 hours prior to admission. Animals were randomly assigned to each group and there was no significant difference in weight, age or temperament between the groups: Medetomidine and buprenorphine (MB) group cats (21/41 cats) received 600 µg/m^2 ^medetomidine and 180 µg/m^2^ buprenorphine. Medetomidine and methadone (MM) group cats (20/41 cats) received 500 µg/m^2 ^medetomidine and 5 mg/m^2^ methadone. Both of these combinations were given by deep intramuscular (IM) injection. Subcutaneous meloxicam was given to every cat at a dose of 3 mg/m^2^ in the MB group and 2.5 mg/m^2 ^in the MM group. Anaesthesia was induced 15 minutes after the premedication with 3 mg/kg alfaxalone IM. Intravenous (IV) catheterisation was only performed in pregnant or ASA 2 patients. A prophylactic IM amoxicillin dose was given 5 minutes after induction. Cats then underwent laryngeal application of lidocaine and endotracheal intubation and responses to this were recorded. Cats were maintained on isoflurane in oxygen via an Ayre’s T-Piece and placed in dorsal recumbency on a heated table. All cats underwent a midline ovariohysterectomy by a single surgeon. The isoflurane was turned off at the end of surgery and oxygen was supplied for a further 5 minutes before the animal was extubated. Atipamezole was given at 50% the medetomidine volume IM. Rescue analgesia was given if pain scoring deemed it necessary.
Study design	Blinded, randomised controlled trial.
Outcome studied	Pain scoring was performed at 10, 20 and 30 minutes postextubation using the simple descriptive scale (SDS), numeric rating scale (NRS), dynamic interactive visual analogue scale (DIVAS), and the UNESP-Botucatu multidimensional composite pain scale systems. These are all subjective pain scoring methods and were carried out by a single, blinded assessor. The results of the pain scoring determined whether rescue analgesia was needed. Heart rate, respiratory rate, mucous membranes, capillary refill time (CRT), temperature, and peripheral pulses were monitored prior to premedication and at 5 minutes intervals afterwards. Intraoperative monitoring of vitals was performed by a blinded individual.
Main findings (relevant to PICO question)	The results of four different pain scales at three time points did not indicate it necessary to administer rescue analgesia for any animal in the trial. The DIVAS pain score was significantly higher in the MB group compared to the MM group at the 10-minute point (P = 0.027). There were no significant differences in pain scores between the MM and MB groups at any time point when using the SDS, NRS or UNESP-Botucatu multidimensional composite pain scale.
Limitations	Mixed population of different ages, weights, and temperaments. The target number of animals to include in the study according to the power calculation was not reached. This could have led to type 1 and 2 statistical errors. Pain scoring was only done up to 30 minutes postoperatively. Only one of the pain scoring systems (UNESP-Botucatu multidimensional composite pain scale) is validated. The spays were carried out midline. The dose of meloxicam given to cats in the MM and MB group was different.

Table note...

**Shah et al. (2019):** 

Population	Entire female cats recruited from the Greater Manchester RSPCA animal hospital. Cats included if they were > 4 months, could be handled, were American Society of Anaesthesiologists (ASA) score 1 or 2, and had not received analgesia, anaesthesia, or sedation within the previous 7 days.
Sample Size	120 cats.
Intervention Details	Cats were divided equally into two groups: 60/120 cats received 600 µg/m^2^ medetomidine, 60 mg/m^2^ ketamine, 3 mg/m^2^ midazolam and 5 mg/m^2^ methadone (intramuscularly) IM. 60/120 cats received 600 µg/m^2^ medetomidine, 60 mg/m^2^ ketamine, 3 mg/m^2^ midazolam and 180 µg/m^2^ buprenorphine IM. Lidocaine was sprayed topically onto the larynx and an endotracheal tube was placed. Anaesthesia was maintained with isoflurane in oxygen, delivered by a T-piece. Depth and physiological parameters were monitored prior to incision, at the time of incision, at the time of pedicle ligation, ligation of cervix, and placement of final skin sutures. A midline ovariohysterectomy was performed by a single surgeon. If pain scoring deemed it necessary, rescue analgesia was given. This was a dose of methadone at 0.5 mg/kg IM. Pain was then assessed 30 minutes later and another dose of methadone 0.5 mg/kg IM was given if needed. All cats received meloxicam 8 hours postoperatively at a dose of 0.3 mg/kg subcutaneously (SC).
Study design	Blinded, randomised, controlled trial.
Outcome studied	Subjective measurement of pain was done through two different pain scoring systems: dynamic interactive visual analogue scale (DIVAS) and Glasgow Feline Composite Measure Pain Scale (CMPS-F). This was performed at 2, 4, 6, and 8 hours after QUAD administration by a single, blinded assessor. A CMPS-F score of over 4/16 indicated the need for rescue analgesia. Heart rate, respiratory rate, sedation, and mechanical nociceptive threshold testing (MNT) were measured once the cat was fully sedated, and then again at 2, 4, 6, and 8 hours after QUAD administration. Physiological parameters were monitored throughout anaesthesia.
Main findings (relevant to PICO question)	Although postoperative CMPS-F scores were not significantly different at any one time between the groups, over time the methadone group had lower CMPS-F scores than the buprenorphine group (P = 0.04). This led to a significant difference in the number of cats requiring rescue analgesia between the groups. In the methadone group 18/60 cats required rescue analgesia, compared to 29/60 cats in the buprenorphine group (P = 0.04). All rescue analgesia was administered in the first 6 hours following QUAD administration. There was no significant difference in DIVAS pain scores between groups over time.
Limitations	Mixed population of cats of different ages and weights. The only validated pain scoring system used was CMPS-F. The other system used was DIVAS which is not validated. In this study the cats did not receive meloxicam until 8 hours after surgery. The assessor was not blinded to treatment group following rescue analgesia. The spays were carried out midline.

Table note...

**Slingsby et al. (2014):** 

Population	A mixture of male and female cats entering the clinic for castration or ovariohysterectomy. All cats were American Society of Anaesthesiologists (ASA) grade 1 or 2 and had not been treated with analgesics, sedative or anesthetic drugs in the previous seven days.
Sample Size	45 cats in total, 24 females and 21 males. Data not relevant to the PICO will not be followed up on.
Intervention Details	Cats were randomly assigned to three premedication groups, there were 8 female cats in each: The methadone group received 20 µg/kg medetomidine and 0.5 mg/kg methadone intramuscularly (IM). The buprenorphine group received 20 µg/kg medetomidine and 20 µg/kg buprenorphine. The butorphanol group received 20 µg/kg medetomidine and 0.4 mg/kg butorphanol. Cats were left undisturbed in a quiet environment for 20 minutes following premedication. Every animal had a 22G intravenously (IV) catheter placed in the cephalic vein and anaesthesia was induced with propofol, given to effect. The cats underwent laryngeal application of lidocaine and an endotracheal tube was placed. Anaesthesia was maintained with isoflurane in oxygen, via a T-piece system. Ovariohysterectomy was carried out by a single experienced surgeon, blinded to the treatment group. Ovariohysterectomy was done via a 2 cm flank incision. If postoperative interactive visual analogue scale (IVAS) scoring deemed it necessary, cats were given rescue analgesia. At 6 hours after premedication all cats received methadone 0.5 mg/kg IM and carprofen 4 mg/kg subcutaneously (SC).
Study design	Assessor-blinded, randomised, controlled trial.
Outcome studied	Subjective assessment of pain which was carried out through IVAS pain scoring. This was done before premedication, just before IV catheterisation and at 1.5, 2, 3, 4, 5, 6, 7, 8, and 20–24 hours postoperatively. Scoring was done by a single assessor who was blinded to the treatment group. The system involved observation from a distance, response to opening cage door, speaking to the animal, and handling the animal. 0 mm indicated no pain and 100 mm indicated the worst pain possible. The results of the IVAS scoring determined the need for rescue analgesia. If a cat scored ≥ 50 mm in the first 6 hours following premedication, rescue analgesia was given. End tidal CO_2 _(ETCO_2_), inspired and expired isoflurane and Oxygen saturation (SpO_2_) were measured by a multi-parameter monitor and recorded every 5 minutes during anaesthesia. Blood pressure was monitored in female cats only via a non-invasive method. Level of sedation using an SDS system, Mechanical Nociceptive Threshold (MNT), rectal temperature, heart rate and respiratory rate were also measured at these time points. These were also measured by a single, blinded assessor.
Main findings (relevant to PICO question)	IVAS pain scores were generally low in the majority of cats in the study. 2/8 cats in the methadone group required rescue analgesia compared to none in the buprenorphine group, but, there was no significant difference between groups in the need for rescue analgesia. When IVAS scores were uncorrected for rescue analgesia, at + 3 hours, pain scores were significantly lower in the buprenorphine group than the methadone group (P < 0.05). When IVAS scores were corrected for rescue analgesia, at + 3, + 4 and + 6 hours, pain scores were significantly higher in the methadone group than the buprenorphine group (P < 0.05).
Limitations	A mixed population of ages and weights of cats. The only pain scoring system used in this study was IVAS, which is not validated. The group receiving buprenorphine may have had lower pain scores in this trial due to its longer duration of action. The carprofen was given 6 hours after premedication. The power analysis calculated that 10 cats were needed in each group (methadone, buprenorphine and butorphanol), and although the total number of animals in each group exceeded this, half of the animals were males undergoing castration meaning there were less than 10 animals undergoing ovariohysterectomy in each group.

Table note...

## Appraisal, application and reflection

The aim of this Knowledge Summary was to critically appraise the existing evidence to determine whether methadone or buprenorphine is a better postoperative analgesic for cats undergoing ovariohysterectomy. A search of the literature returned four papers that were relevant to the PICO (Shah et al., 2019; Mahdmina et al., 2020; Slingsby et al., 2014; Bortolami et al., 2013). All four papers were assessor-blinded randomised controlled trials that directly compared postoperative pain scores in cats receiving buprenorphine or methadone. Two of the studies (Bortolami et al., 2013; Slingsby et al., 2014) also compared butorphanol which will not be discussed further in this Knowledge Summary. The same two studies also had male cats in the treatment groups; however, because the pain scores from the males and females were presented separately, it was possible to just analyse the pain scores from female cats undergoing ovariohysterectomy.

All studies reported that both buprenorphine and methadone provided adequate analgesia for ovariohysterectomy in cats; however, some papers reported significant differences in pain scores postoperatively. Shah et al. (2019) reported that although Glasgow Composite Measure Pain Scale – Feline (CMPS-F) scores were not significantly different at any one time, over time the cats receiving methadone had a significantly lower CMPS-F score compared to the cats receiving buprenorphine. This led to significantly more rescue analgesia in the group receiving buprenorphine. Mahdmina et al. (2020) also found methadone to have an advantage over buprenorphine as dynamic interactive visual analogue scale (DIVAS) pain scores were significantly higher in the group receiving buprenorphine than the group receiving methadone 10-minutes postsurgery. However, there were no significant differences in the three other pain scores used or at any other time point. In contrast, Slingsby et al. (2014) found that buprenorphine was associated with lower postoperative interactive visual analogue scores (IVAS) than methadone at 3, 4, and 6 hours after premedication when pain scores were corrected for rescue analgesia. Finally, Bortolami et al. (2013) found no significant differences in IVAS pain scores between methadone and buprenorphine at any time after premedication.

In all studies the person pain scoring the cats was the same and was blinded to treatment group, however the pain scoring methods utilised differed between studies. The two older studies (Bortolami et al., 2013; Slingsby et al., 2014) used IVAS pain scoring. IVAS pain scoring is an unvalidated system and the scores are often highly variable. In comparison, the two newer studies (Mahdmina et al., 2020; Shah et al., 2019) both used a combination of validated and unvalidated pain scoring systems with Mahdmina et al. (2020) using simple descriptive scale (SDS), numeric rating scale (NRS), DIVAS and UNESP-Botucatu multidimensional composite pain scale and Shah et al. (2019) using DIVAS and CMPS-F. Even though the validated systems are still subjective measures of pain, they have been tested to confirm their validity, sensitivity, and reliability, and therefore the results can be relied upon far more than the non-validated systems (Steagall and Monteiro, 2019). Therefore, the studies in this Knowledge Summary that use validated scoring systems provide stronger evidence (Mahdmina et al., 2020; Shah et al., 2019), and the findings are more reliable than the studies that do not (Bortolami et al., 2013; Slingsby et al., 2014).

Even when using validated scoring systems, pain assessment in cats presents many challenges. Animals that have a shy, fearful, or aggressive demeanor are often given higher pain scores. This can be a problem in a hospital environment where cats often present with behavioral changes because they are stressed (Steagall & Monteiro, 2019). Furthermore, it is challenging to differentiate between pain and dysphoria. This is particularly relevant in this Knowledge Summary as patients recovering from anaesthesia who have received opioids can often be very dysphoric. This would lead to an inflation of pain scores. This raises the question as to whether pain scoring is a sensitive enough tool to evaluate the efficacy of analgesia, and whether more objective measures such as mechanical nociceptive threshold testing would provide a more reliable conclusion.

The other main thing that varied between these studies was at what point the NSAID was administered. In general practice the NSAID is usually given at the time of premedication or intraoperatively as this provides multimodal analgesia, which has been shown to be more effective (Steagall et al., 2022). However, in these studies there was a judgement to be made on whether to give the NSAID later so that any differences in pain scores between the methadone and buprenorphine were more likely to be identified or give it at the same time as the premedication because it would be easier to extrapolate the results of the study to a general practice situation. Mahdmina et al. (2020) was the only study reviewed that gave the NSAID at the time of premedication. The other studies (Shah et al., 2019; Bortolami et al., 2013; Slingsby et al., 2014) either gave the NSAID at 6 or 8 hours after premedication. This means that the results of these studies may be less comparable to general practice. Moreover, in the study by Mahdmina et al. (2020) the doses of meloxicam given to the cats in the methadone and buprenorphine groups were different with the cats who received methadone getting 3 mg/m^2^.

On the other hand, the studies undertaken by Bortolami et al.(2013) and Slingsby et al.(2014) may be more comparable to general practice because they performed flank ovariohysterectomies in the study, Shah et al. (2019) and Mahdmina et al. (2020) both performed midline ovariohysterectomies. In the UK the majority of cats are neutered via the flank approach as it is less invasive and associated with fewer wound infections (Munif, Safawat & Abdul, 2022). Flank ovariohysterectomies are associated with a greater level of pain than midline ovariohysterectomies because the incision is made through muscle tissue rather than the linea alba. Muscle has more nociceptors that connect to Aδ and C fibres than the linea alba which results in more nociception and pain (Grint et al., 2006). This may limit how easy it is to extrapolate the results of the studies that performed midline ovariohysterectomies to UK general veterinary practice. Unfortunately, these studies are also the ones that used validated pain scoring methods. Therefore, to draw conclusions that were more useful for UK first opinion practice, more studies using the validated pain scoring methods would be needed where flank ovariohysterectomies were performed.

Sample size targets were calculated in every study; however, these were not always reached. Mahdmina et al. (2020) calculated that 50 cats were needed; however, only 41 cats finished the study. In Shah et al. (2019), the necessary sample size was calculated to be 58, which was exceeded, as 120 cats were recruited and all cats finished the study. In both the Bortolami et al. (2013) and Slingsby et al. (2014) the target sample sizes were both 30, and the actual number of cats in each study was 45. However, both studies also included male cats, meaning the number of female cats in each study was only 24. This means that not only were these studies underpowered for this Knowledge Summary, but considering males had low pain scores and were deemed a poor surgical model, it also means that this study in general was underpowered to detect differences between groups. It is possible that the lack of significant difference in pain scores between the cats receiving methadone and the cats receiving buprenorphine was actually due to there not being enough cats in the studies, leading to a type II statistical error, rather than there not being a difference in analgesia provided. Interestingly, the study with the most consistent differences in pain scores over the whole postoperative period (Shah et al., 2019) was also the study that massively exceeded the sample size predicted by the power calculation. This indicates the need for more research using bigger sample sizes.

The length of time that cats were pain scored for postoperatively varied between studies. One study only recorded pain scores up to 30 minutes postextubation to determine whether rescue analgesia was needed (Mahdmina et al., 2020). This may have been too soon after extubation to allow the cat to recover and start showing signs of pain, because no cats in this study required rescue analgesia. The 2022 International Society of Feline Medicine (ISFM) consensus guidelines on management of acute pain suggest waiting for 45 minutes postoperatively and continuing pain assessments until discharge (Steagall et al., 2022). By pain scoring for a longer period postoperatively it would have given a better assessment of the duration of action and efficacy of the two opioids. The other three studies monitored pain scores up to 8 hours after premedication to determine whether rescue analgesia was needed (Shah et al., 2019; Bortolamiet al., 2013; Slingsby et al., 2014). In all these studies some patients required rescue analgesia which demonstrates the importance of pain scoring for a longer duration postoperatively.

In all studies the cats were of mixed ages, however the mean age was not significantly different between the groups receiving buprenorphine or methadone. This ensures that age was not a confounding factor in the studies reviewed. Table 1 below gives the mean age of cats for each of the studies. By studying a mix of ages it also means that any conclusions that we can draw assume that the efficacy of analgesia is the same in cats of all ages. However, research has shown that more frequent opioid dosing may be necessary in young cats. One study showed that the age of the cat can affect thermal antinociception produced by opioids (Steagall et al., 2022). This is particularly noteworthy because the recommended age of neutering in cats is from 4 months (The Cat Group, 2006) and many of the studies reviewed here have mean ages greater than 4 months (see table 1). This may limit how well the results of these studies can be extrapolated to UK veterinary practice. Therefore, more studies are warranted to see whether there may be a difference in analgesic efficacy, between buprenorphine and methadone, in specific age groups.

**Table 1:** Average age in months of the cats in each study. Where the study gave the ages separately for each opioid group, this is specified in the table.

Study	Mean and range of ages of cats in the study (months)
Shah et al. (2019)	Buprenorphine group: 16.2 ± 14.4 Methadone group: 13.3 ± 9.46
Mahdmina et al. (2020)	Buprenorphine group: 12 (3–48) Methadone group: 14 (2–48)
Bortolami et al. (2013)	7 ± 4 (not presented by opioid group)
Slingsby et al. (2014)	8.7 ± 5.1 (not presented by opioid group)

Table note...

The current evidence demonstrates that both buprenorphine and methadone provide adequate analgesia in cats undergoing ovariohysterectomy. In the studies that used validated pain scoring methods (Mahdmina et al., 2020; Shah et al., 2019) and therefore, the more reliable studies, methadone was found to be associated with lower pain scores overall, or at selected time points postoperatively. Therefore, it can be concluded that there is moderate evidence to suggest methadone is a better analgesic for feline ovariohysterectomy than buprenorphine. Future research should focus on making use of the three validated pain scoring systems that are now available and use large enough sample sizes. To be able to better extrapolate the results to UK veterinary practice it would also be useful to have more studies focusing on flank ovariohysterectomies in cats around 16 weeks of age. Ultimately this Knowledge Summary highlights the importance of using validated postoperative pain scoring in general practice to identify cats who require additional analgesia.

## Methodology

**Search strategy:** 

Databases searched and dates covered	CAB Abstracts on CABI Digital library – January 1973 to January 2024 PubMed via NCBI interface – January 1970 to January 2024 Web of Science core collections on Web of Science – 1900 January 2024
Search terms	CAB Abstracts: (cat OR cats OR feline OR felines OR felid*) (Neuter* OR ovariohysterect* OR spay* OR spey*) Methadone Buprenorphine (Analgesia OR analgesic) 1 AND 2 AND 3 AND 4 AND 5 Web of science core collections: (cat OR cats OR feline OR felines OR felid*) (Neuter* OR ovariohysterect* OR spay* OR spey*) Methadone Buprenorphine (Analgesia OR analgesic) 1 AND 2 AND 3 AND 4 AND 5 PubMed: (cat OR cats OR feline OR felines OR felid*) AND (neuter* OR ovariohysterect* OR spay* OR spey*) AND (buprenorphine) AND (methadone) AND (analgesia OR analgesic)
Dates searches performed:	06 Jan 2024

Table note placeholder

**Exclusion / inclusion criteria:** 

Exclusion	Studies not relevant to the PICO. Studies not written in English. Review papers. Conference proceedings. Case reports with only one cat.
Inclusion	Primary research studies relevant to the PICO, that were peer reviewed. Papers that assessed pain using pain scores.

Table note placeholder

**Search outcome:** Caption placeholder

Database	Number of results	Excluded – not relevant to PICO	Excluded – conference paper	Excluded – duplicates	Total relevant papers
CAB Abstracts	7	2	1	0	4
Web of Sciencecore collection	18	13	1	0	4
Pubmed	6	1	0	1	4
Total relevant papers when duplicates removed					4

Table note placeholder

## ORCiD

Isobel Lawrence: https://orcid.org/0009-0009-6087-7043

## Conflict of interest

The author declares no conflicts of interest.

## Disclaimer

Knowledge Summaries are a peer-reviewed article type which aims to answer a clinical question based on the best available current evidence. It does not override the responsibility of the practitioner. Informed decisions should be made by considering such factors as individual clinical expertise and judgement along with patient’s circumstances and owners’ values. Knowledge Summaries are a resource to help inform and any opinions expressed within the Knowledge Summaries are the author’s own and do not necessarily reflect the view of the RCVS Knowledge. Authors are responsible for the accuracy of the content. While the Editor and Publisher believe that all content herein are in accord with current recommendations and practice at the time of publication, they accept no legal responsibility for any errors or omissions, and make no warranty, express or implied, with respect to material contained within. For further information please refer to our Terms of Use.
